# The Effectiveness of Virtual Reality Exposure–Based Cognitive Behavioral Therapy for Severe Anxiety Disorders, Obsessive-Compulsive Disorder, and Posttraumatic Stress Disorder: Meta-analysis

**DOI:** 10.2196/26736

**Published:** 2022-02-10

**Authors:** Inge van Loenen, Willemijn Scholten, Anna Muntingh, Johannes Smit, Neeltje Batelaan

**Affiliations:** 1 GGZ inGeest Specialized Mental Health Care Amsterdam Netherlands; 2 Amsterdam UMC, Vrije Universiteit Amsterdam, Psychiatry, Amsterdam Public Health Research Institute Amsterdam Netherlands

**Keywords:** anxiety disorders, virtual reality, virtual reality exposure therapy, cognitive behavioral therapy, meta-analysis, mobile phone

## Abstract

**Background:**

In recent years, virtual reality exposure–based cognitive behavioral therapy (VRE-CBT) has shown good treatment results in (subclinical) anxiety disorders and seems to be a good alternative to exposure in vivo in regular cognitive behavioral therapy (CBT). However, previous meta-analyses on the efficacy of VRE-CBT on anxiety disorders have included studies on specific phobias and subthreshold anxiety; therefore, these results may not be generalizable to patients with more severe and disabling anxiety disorders.

**Objective:**

The objective of our study is to determine the efficacy of VRE-CBT on more severe anxiety disorders, excluding specific phobias and subthreshold anxiety disorders. Meta-analyses will be conducted to examine the efficacy of VRE-CBT versus waitlist and regular CBT. Our secondary objectives are to examine whether the efficacy differs according to the type of anxiety disorder, type of recruitment, and type of VRE-CBT (virtual reality exposure either with or without regular CBT). Furthermore, attrition in VRE-CBT and CBT will be compared.

**Methods:**

Studies published until August 20, 2020, were retrieved through systematic literature searches in PubMed, PsycINFO, and Embase. We calculated the effect sizes (Hedges *g*) for the difference between the conditions and their 95% CIs for posttest and follow-up measurements in a random effects model. A separate meta-analysis was performed to compare attrition between the VRE-CBT and CBT conditions.

**Results:**

A total of 16 trials with 817 participants were included. We identified 10 comparisons between VRE-CBT and a waitlist condition and 13 comparisons between VRE-CBT and a CBT condition. With regard to risk of bias, information on random sequence generation, allocation concealment, and risk of bias for selective outcome reporting was often absent or unclear. The mean effect size of VRE-CBT compared with waitlist (n_co_=10) was medium and significant, favoring VRE-CBT (Hedges *g*=−0.490, 95% CI −0.82 to −0.16; *P*=.003). The mean effect size of VRE-CBT compared with CBT (n_co_=13) was small and nonsignificant, favoring CBT (Hedges *g*=0.083, 95% CI −0.13 to 0.30; *P*=.45). The dropout rates between VRE-CBT and CBT (n_co_=10) showed no significant difference (odds ratio 0.79, 95% CI 0.49-1.27; *P*=.32). There were no indications of small study effects or publication bias.

**Conclusions:**

The results of our study show that VRE-CBT is more effective than waitlist and as effective as CBT in the treatment of more severe anxiety disorders. Therefore, VRE-CBT may be considered a promising alternative to CBT for patients with more severe anxiety disorders. Higher-quality randomized controlled trials are needed to verify the robustness of these findings.

## Introduction

### Background

Exposure-based cognitive behavioral therapy (CBT) is known as the *gold standard* for treatment of patients with anxiety disorders as its efficacy has been well-established by extensive research [1,2]. Despite its efficacy, potential disadvantages concern CBT being time- and cost-inefficient, impracticable, or considered too aversive by patients [3]. Virtual reality exposure–based cognitive behavioral therapy (VRE-CBT) offers an alternative to regular CBT and has several advantages. It allows immersion within a feared virtual environment that is tailored to the individual patient while being offered within a convenient and safe clinical setting. In VRE-CBT, the therapist can ensure complete control over the content and dose of feared stimuli and therefore optimize individualized pacing through exposures. Moreover, each step of VRE-CBT can be repeated as often as needed before proceeding to the next feared situation, thereby facilitating the complex processes responsible for the effect of exposure therapy. Attrition rates between VRE-CBT and CBT do not seem to differ [4]. However, it was shown that most of a clinical sample with phobic disorders (76%) preferred VRE-CBT over exposure in vivo, and refusal rates of those who were offered VRE-CBT (3%) were substantially lower compared with refusal rates of those who were offered exposure in vivo (27%) [5].

In recent years, many studies have been conducted on the efficacy of VRE-CBT on anxiety disorders and have shown good treatment results. A total of 5 meta-analyses on the efficacy of VRE-CBT versus control conditions, consisting mostly of studies on specific phobias, have been published. They consistently show a clear superiority of VRE-CBT versus nonactive control groups (ie, waitlist) [6-10] and similar [6,7,9,10] or larger [8] effects of VRE-CBT versus CBT conditions incorporating in vivo exposure. In addition, Opris et al [6] showed good stability of VRE-CBT results over time, similar to those of CBT, and a dose-response relationship with more sessions yielding a larger treatment effect. Although it has been questioned whether VRE-CBT induces significant behavior changes in real life [8], Morina et al [7] showed that treatment gains after VRE-CBT generalize well to real-life situations as measured by means of behavioral laboratory tests and recordings of behavioral activities in real life [7]. Considering the advantages of VRE-CBT and its promising results in previous meta-analyses, it is not surprising that a significant expansion of VRE-CBT in mental health care is both predicted and advocated to occur in the coming years [11]. However, these meta-analyses have limited generalizability to patients with more severe and disabling anxiety symptoms—a diagnosis of an anxiety disorder according to the criteria of the Diagnostic and Statistical Manual of Mental Disorders, Fourth and Fifth Edition (DSM-IV and DSM-V, respectively), and the International Classification of Diseases, 10th and 11th Revision (ICD-10 and ICD-11, respectively), established by a structured diagnostic interview, excluding specific phobias. First, most previous meta-analyses included studies that completely [7], largely, or partly [6,8-10] focused on specific phobias. However, specific phobias are generally less severe than more disabling anxiety disorders such as social anxiety disorder (SAD), posttraumatic stress disorder (PTSD), panic disorder (PD), and agoraphobia (AGO). Second, the inclusion of studies in previous meta-analyses was not restricted to samples with a formal anxiety disorder diagnosis (following DSM or ICD criteria), thereby including subthreshold anxiety disorders that are less severe. Carl et al [9] also stated that “The applications for clinical settings are clearer when conclusions can be drawn from clinical samples.” The experience with and effects of VRE-CBT may be different for patients with more severe symptoms. On the one hand, they might be more reluctant to expose themselves to feared situations with regular CBT and may favor and benefit most from a controlled exposure therapy setting, such as in VRE-CBT. On the other hand, treating these more severe anxiety disorders with VRE-CBT may be far more challenging than treating milder symptoms. VRE-CBT for the treatment of more severe anxiety disorders requires more varied and elaborate virtual settings while still ensuring a high sense of presence (the feeling that one is actually present in the virtual world). For example, rather than fear of a specific situation or object, treatment of AGO generally requires exposure to various public places (eg, streets, shops, and public transport). In addition, for SAD, it may be essential that VRE-CBT also incorporates interaction through verbal and nonverbal behavior of others in response to the behavior of the patient as SAD centers on the perception of negative evaluation. In addition to the predominance of studies targeting specific phobias, most previous meta-analyses also included nonrandomized studies, thereby possibly leading to biased results. Ultimately, there is a lack of evidence for the efficacy of VRE-CBT in the treatment of more severe and formal anxiety disorders. Hence, a careful examination of research is necessary to prevent unjustified expansion of VRE-CBT in clinical settings.

We conducted a meta-analysis to determine whether the positive results of VRE-CBT as reported in earlier meta-analyses are sustained when focusing solely on more severe anxiety disorders (according to DSM-IV, DSM-V, ICD-10, or ICD-11 criteria): PD with or without AGO, AGO, SAD, generalized anxiety disorder (GAD), obsessive-compulsive disorder (OCD), or PTSD, limiting our inclusion criteria to studies with a formal diagnosis to avoid heterogeneity in our sample.

### Objectives

Our main objective is to examine the efficacy of VRE-CBT on anxiety severity compared with (1) waitlist and (2) CBT at posttest and follow-up measurements (if available). Our secondary objectives are to examine whether efficacy differs according to the type of anxiety disorder, type of recruitment, and type of VRE-CBT (virtual reality exposure [VRE] with or without regular CBT). Furthermore, the feasibility of VRE-CBT has been evaluated by comparing the attrition of VRE-CBT and CBT.

## Methods

### Study Selection

Studies published until August 20, 2020, were retrieved through systematic literature searches in PubMed, PsycINFO, and Embase (see Multimedia Appendix 1 for the complete search strings) for studies that randomized patients with anxiety disorders into VRE-CBT, active control condition, or an inactive control condition and compared the effects of these treatments. A librarian and IvL conducted the search by combining search terms indicative of anxiety disorders (PD with or without AGO, AGO, GAD, SAD, OCD, and PTSD) with terms indicative of VRE therapy. Furthermore, we checked the reference lists of the retrieved articles and previous meta-analyses and reviews for additional studies.

The inclusion criteria were (1) adult patients (aged >18 years); (2) at least one VRE-CBT condition; (3) random assignment to conditions; (4) comparison with waitlist or CBT without virtual reality (VR); (5) measure of outcome related to anxiety; (6) a primary diagnosis of an anxiety disorder according to DSM-IV, DSM-V, ICD-10, or ICD-11 criteria established by a structured diagnostic interview, excluding specific phobias; and (7) original empirical findings. Consistent with the DSM-IV or DSM-V classification, PTSD and OCD were also included even though these are no longer classified as anxiety disorders in the DSM-V. No date restrictions were applied. Articles consisting of only abstracts or not presenting original data were excluded, as were studies with a crossover design. No language restrictions were applied.

In total, 2 authors (WS and IvL) independently assessed the list of titles and abstracts that resulted from the literature search for eligibility. WS and IvL independently examined full texts and selected eligible randomized controlled trials (RCTs). Discrepancies were resolved by consulting and discussing with a third author (NB). This meta-analysis was conducted in accordance with the PRISMA (Preferred Reporting Items for Systematic Reviews and Meta-Analyses) guidelines [12].

### Data Extraction

In total, 2 authors (WS and IvL) independently extracted the data from the selected articles. This information included characteristics of the study population (mean age and range and gender), classification instrument, recruitment method, primary outcome, and conditions (sample size, number of dropouts, number of sessions, and follow-up in months for each condition).

For studies using more than one validated outcome measure, we selected the reported primary outcome measure. When undefined, we selected the domain-specific outcome that was most frequently used in the included trials for that type of anxiety disorder as the primary outcome measure.

For each study, the mean and SD of the primary outcome on posttest and follow-up measurements were used to calculate the effect sizes. For the study of Pitti et al [13], the effect size was calculated using the mean and *P* value of the between-group comparison as SDs were not available. Discrepancies in data extraction were resolved through discussion and by studying the original article. In case of a lack of clarity or missing data, the authors of selected studies were contacted.

### Risk of Bias Assessment

In total, 2 authors (WS and IvL) independently assessed the risk of bias in the included studies. Differences were resolved through discussion. Five areas of risk of bias according to the Cochrane tool for assessing risk of bias were rated [14]: (1) random sequence generation, (2) allocation concealment, (3) blinding of outcome assessment, (4) incomplete outcome data, and (5) selective reporting. The criterion of blinding of participants and personnel was omitted as blinding is not possible in these psychological interventions. We assessed all areas as having low, high, or unclear risk of bias. Incomplete outcome data were assessed by evaluating attrition rates in all the comparison groups. Attrition was defined as all randomized patients who dropped out of the treatment. If attrition rates were >30% and no appropriate methods were used to minimize biased comparisons among groups (intention-to-treat analyses), the study was coded as being at high risk of bias for incomplete outcome data. Selective outcome reporting was assessed by evaluating differences in primary outcomes between publication and trial registration, if available. If no trial registration was available for a study, the study was coded as being at unknown risk for selective outcome reporting. We computed a summary score for each study by summing the number of items scoring high risk of bias, which could range from 0 to 5, with low scores indicating low risk of bias.

### Meta-analysis

Meta-analyses were conducted to assess the effect of VRE-CBT on the severity of anxiety symptoms when compared with waitlist and CBT conditions. We calculated the effect sizes (Hedges *g*) for the difference between the conditions and their 95% CIs for posttest and follow-up measurements if data were available. For the follow-up date, we chose the longest follow-up period available. The effect sizes were based on intention-to-treat analysis, if available. Otherwise, completer analysis results were used. Heterogeneity among the studies was examined using the Cochran *Q* test and Higgins *I*^2^ statistics [15]. The possibility of publication bias was evaluated by visual inspection of the funnel plot and Egger test [16] and using the Duval and Tweedie trim and fill procedure [17]. Random effects models were used as heterogeneity across the studies was expected, for example, because of variations in the number of sessions, treatment protocols, recruitment setting, and type of anxiety disorder.

Predefined subgroup analyses were conducted for type of anxiety disorder and method of recruitment (community, clinical, or both). Meta-regression analyses were used to estimate the impact of year of publication, quality of the individual studies (risk of bias), and treatment duration (in sessions). We conducted sensitivity analyses excluding outliers and studies in which the VRE-CBT condition included additional CBT elements or sessions with psychoeducation, cognitive restructuring, interoceptive exposure, and exposure in vivo. A separate meta-analysis was performed to compare the dropouts between the VRE-CBT and CBT conditions to assess the feasibility of VRE-CBT. Odds ratios indicated the odds of participants dropping out from the VRE-CBT versus CBT. The analyses were performed using Comprehensive Meta-Analysis (version 2.2; Biostat Inc) [18].

## Results

### Study Inclusion

**Figure 1:**
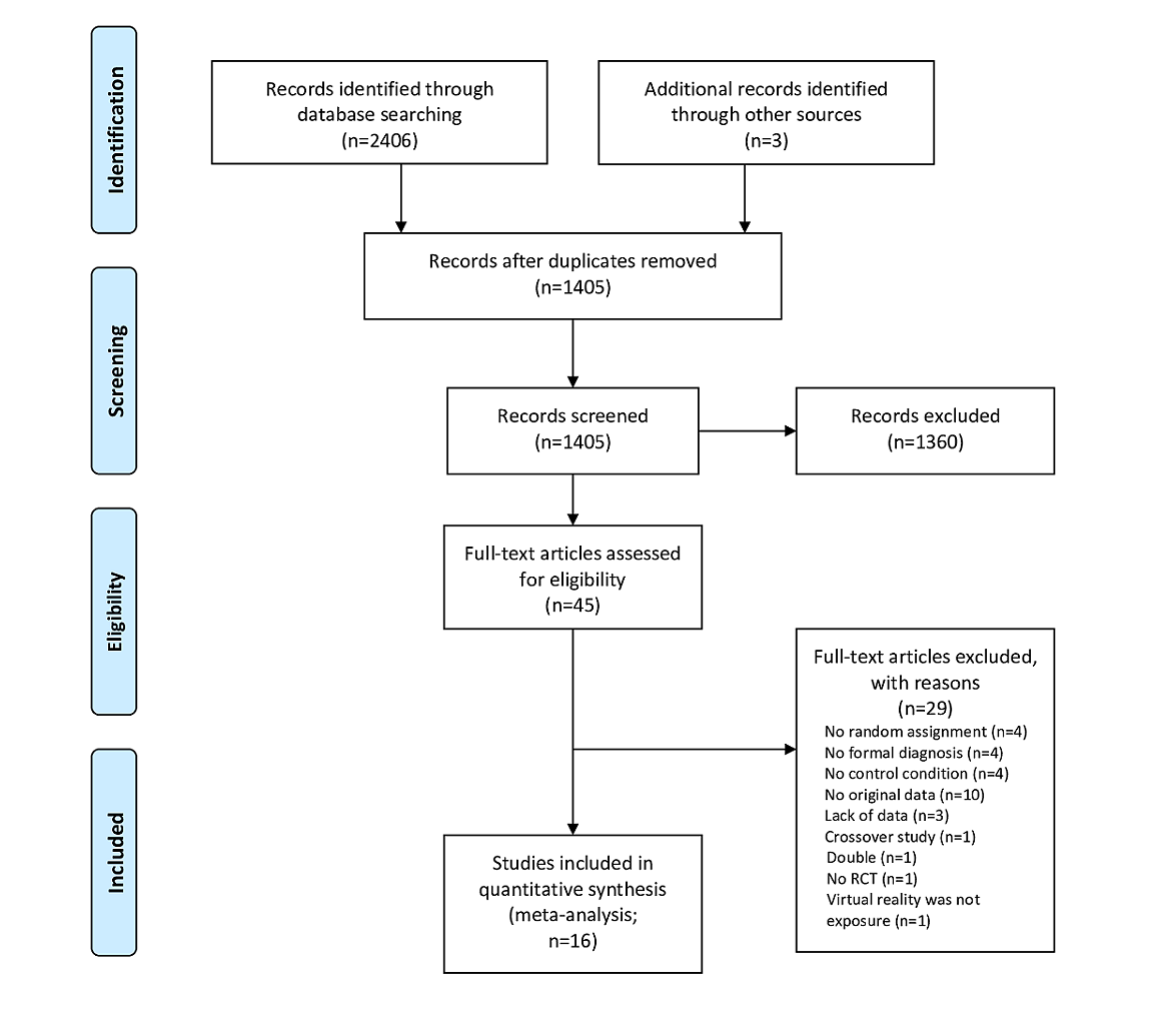
Flowchart of study inclusion. RCT: randomized controlled trial.

After removal of duplicates, the literature searches resulted in a total of 1405 papers for consideration. A total of 2 papers (2/1405, 0.1%) were identified from checking references. After screening the titles and abstracts, of the 1405 papers, 45 (3.2%) full-text articles were retrieved for a more detailed evaluation of eligibility. Figure 1 shows the flowchart of the inclusion process following the PRISMA guidelines [19]. Subsequently, of the 45 articles, 26 (58%) were excluded as they did not meet the inclusion criteria for various reasons. In addition, 3 studies (3/45, 7%) were excluded because of missing data for analyses despite efforts to acquire these data by contacting the authors (for specific reasons, see Figure 1). Finally, 16 studies were included in the meta-analysis.

### Study Characteristics

A total of 16 trials were included in Multimedia Appendix 2 [13,20-34]. These trials entailed 10 comparisons between VRE-CBT and a waitlist and 13 comparisons between VRE-CBT and a CBT condition. The number of participants per study ranged from 10 to 162 (12/16, 75% of the studies had <25 participants in each arm). Follow-up data were available for 38% (5/13) of the comparisons between VRE-CBT and a CBT condition and ranged from 6 to 12 months. Of the 16 studies, 7 (44%) focused on PD with or without AGO, 4 (25%) focused on SAD, 4 (25%) focused on PTSD, and 1 (6%) focused on GAD. None of the screened studies on OCD were included as they did not meet our inclusion criteria.

### Risk of Bias

**Figure 2:**
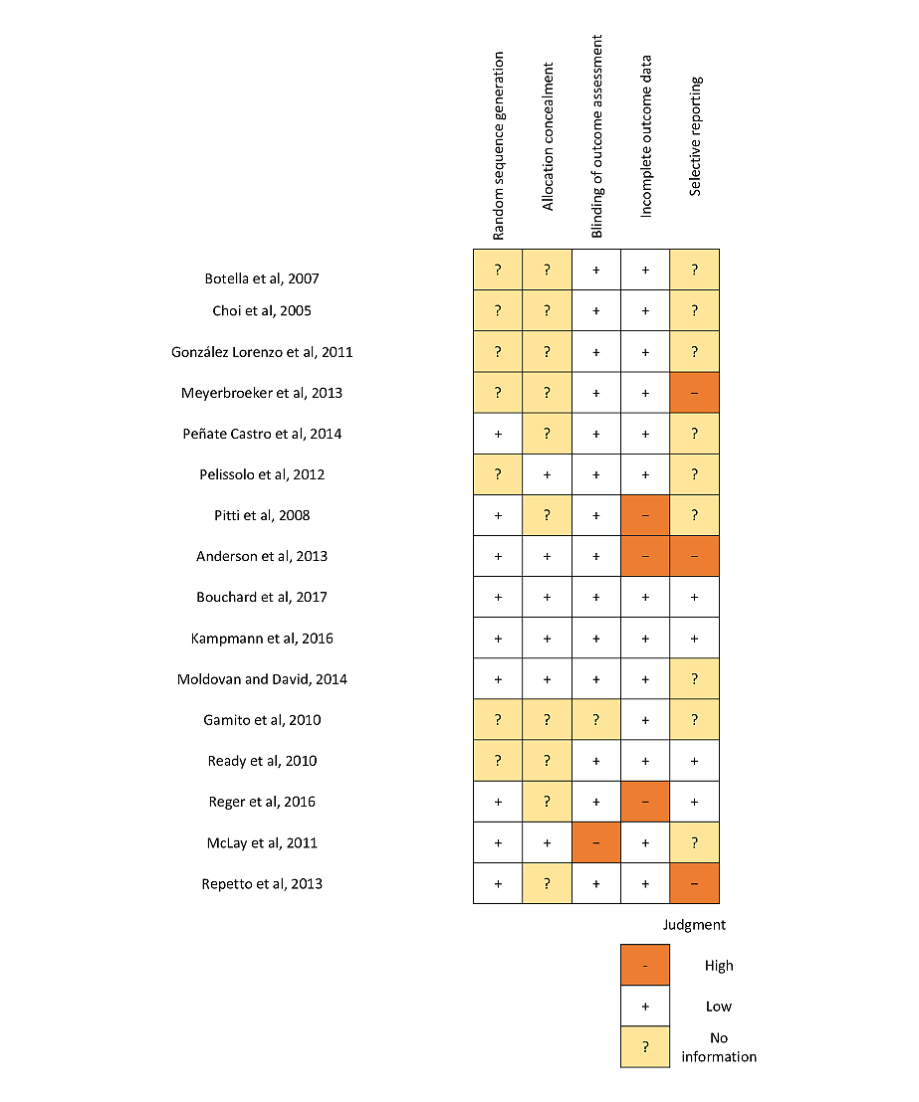
Risk of bias summary [13,20-34].

In each trial, 5 areas of risk of bias were rated as low, unclear (because of lack of information), or high risk of bias. As blinding of participants and personnel was not possible, all studies had a high risk of performance bias; therefore, this was not reported (see Figure 2 for the risk of bias summary). Information on random sequence generation and allocation concealment was absent or not reported clearly in 44% (7/16) and 63% (10/16) of the trials, respectively. Of the 16 trials, 9 (56%) were scored with unclear risk of bias for selective outcome reporting as registration in a trial database could not be found.

### Effect Size: VRE-CBT Versus Waitlist at Posttest Measurement

**Figure 3:**
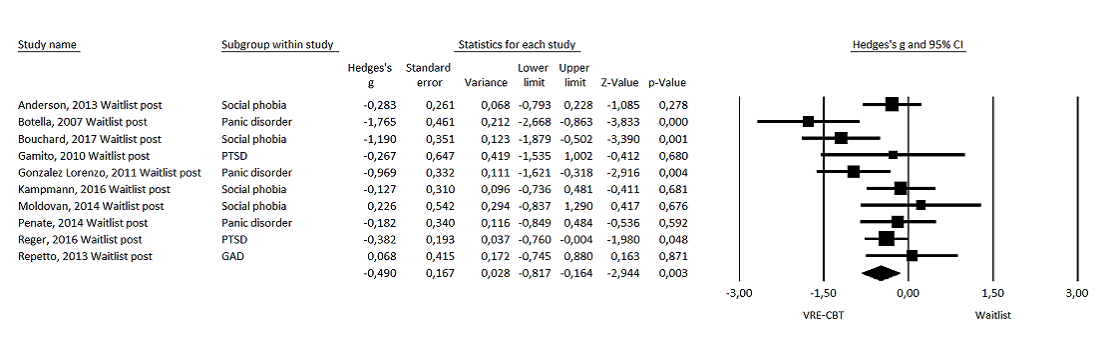
Forest plot of virtual reality exposure–based cognitive behavioral therapy versus waitlist at posttest measurement [20,22,24,26-30,32,34]. GAD: generalized anxiety disorder; PTSD: posttraumatic stress disorder; VRE-CBT: virtual reality exposure–based cognitive behavioral therapy.

The mean effect size of VRE-CBT on symptoms of anxiety when compared with waitlist control at posttest measurement (n_co_=10) was medium and significant, favoring VRE-CBT (Hedges *g*=−0.490, 95% CI −0.82 to −0.16; *P=*.003; see Figure 3 for the forest plot). There was moderate heterogeneity (*I*^2^=56%). Effect size decreased after exclusion of 1 potential outlier [20] (Hedges *g*=−0.391, 95% CI −0.66 to −0.12; *P*=.005), with low heterogeneity (*I*^2^=33%). Sensitivity analysis excluding studies in which VRE-CBT included additional CBT elements or sessions (n_co_=6) showed a decrease in the effect size to Hedges *g* of −0.255 (95% CI −0.49 to −0.02; *P*=.03). Sensitivity analysis excluding 1 study with medication (antidepressants; n_co_=9) as part of the intervention and control conditions showed a decrease in the effect size to Hedges *g* of −0.432 (95% CI −0.78 to −0.09; *P*=.01).

### Effect Size: VRE-CBT Versus CBT at Posttest Measurement

**Figure 4:**
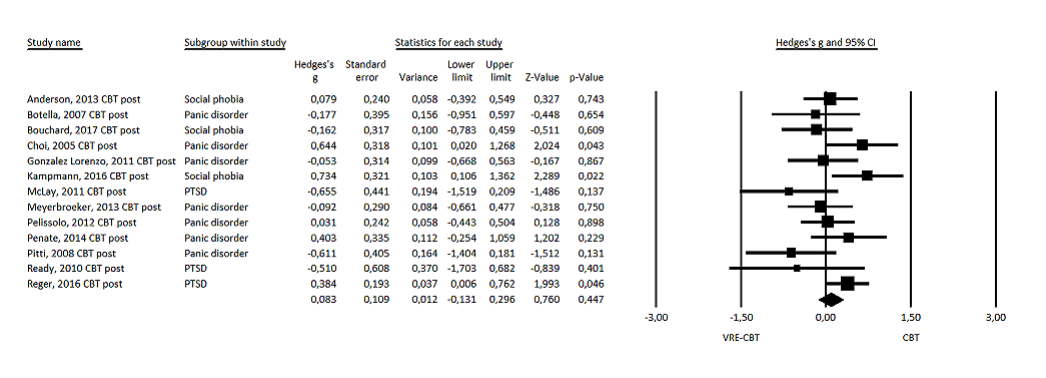
Forest plot of virtual reality exposure–based cognitive behavioral therapy versus cognitive behavioral therapy at posttest measurement [21-28,31-33]. CBT: cognitive behavioral therapy; PTSD: posttraumatic stress disorder; VRE-CBT: virtual reality exposure–based cognitive behavioral therapy.

The mean effect size of VRE-CBT on symptoms of anxiety when compared with CBT at posttest measurement (n_co_=13) was small and nonsignificant, favoring CBT (Hedges *g*=0.083, 95% CI −0.13 to 0.30; *P*=.45) with low to moderate heterogeneity (*I*^2^=36%; see Figure 4 for the forest plot). Sensitivity analysis excluding studies in which VRE-CBT included additional CBT elements or sessions (n_co_=6) showed a nonsignificant increase in the effect size to Hedges *g* of 0.209 (95% CI −0.14 to 0.56; *P*=.24). Sensitivity analysis excluding 1 study with medication (antidepressants; n_co_=12) as part of the intervention and control conditions showed a similar effect size of Hedges *g* of 0.09 (95% CI −0.14 to 0.33; *P*=.34).

### Effect Size: VRE-CBT Versus CBT Condition at Follow-up (6-12 Months)

In total, there were 5 studies (5/16, 31%) with follow-up assessments that were compared with CBT. Of the 16 studies, 3 (19%) only conducted follow-up assessments after 6 months [24,27,31], 1 (6%) had 6- and 12-month assessments [25], and only 1 (6%) had a 12-month follow-up [20]. For the meta-analysis, the longest follow-up period was used. The mean effect size of VRE-CBT on symptoms of anxiety when compared with CBT (n_co_=5) remained nonsignificant at follow-up (Hedges *g*=−0.082, 95% CI −0.40 to 0.24; *P*=.61; *I*^2^=0).

### Subgroup Analyses and Meta-Regression Analyses

Subgroup analyses were performed for the type of anxiety disorder and type of VRE-CBT (VRE either with or without regular CBT). Type of anxiety disorder (SAD: n_co_=3; PD with or without AGO, or AGO: n_co_=7; and PTSD: n_co_=3) was a significant moderator (*P*=.005) for the comparison between VRE-CBT and waitlist, although this result was most likely affected by the high heterogeneity within 2 of the small subgroups—PD with or without AGO, or AGO (*I*^2^=75%) and SAD (*I*^2^=59%). Only 1 GAD study (1/16, 6%) was available; therefore, we did not include this study in the analysis.

We were not able to conduct planned subgroup analyses for type of recruitment (community, clinical, or both) as there was only 1 study (1/16, 6%) with community recruitment, which did not allow for subgroup analysis.

Meta-regression to estimate the impact of year of publication (*P*=.16), treatment duration (in sessions; *P*=.85), and quality of the individual studies (risk of bias; *P*=.32) showed no significant effects in the comparison of VRE-CBT with waitlist control. In the comparison with CBT, year of publication (*P*=.44), treatment duration (in sessions; *P*=.07), and quality of the individual studies (risk of bias; *P*=.50) also showed no significant differences.

### Comparison of Attrition Between VRE-CBT and CBT

A separate meta-analysis was conducted to compare the dropouts between VRE-CBT and CBT conditions to assess the feasibility of VRE-CBT. The dropout rates between the 2 conditions (n_co_=10) showed no significant difference (odds ratio 0.79, 95% CI 0.49-1.27; *P*=.32).

### Publication Bias

**Figure 5:**
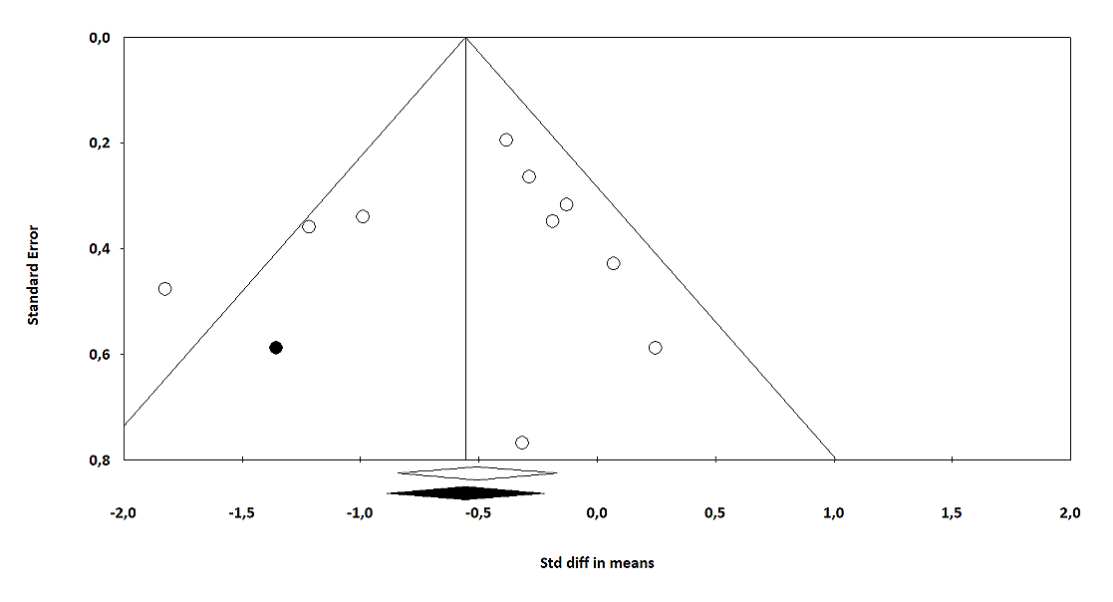
Trim and fill adjusted funnel plot of virtual reality exposure–based cognitive behavioral therapy versus waitlist (the white circles represent the observed studies, and the black circles represent the imputed studies). Std diff: Standard difference.

**Figure 6:**
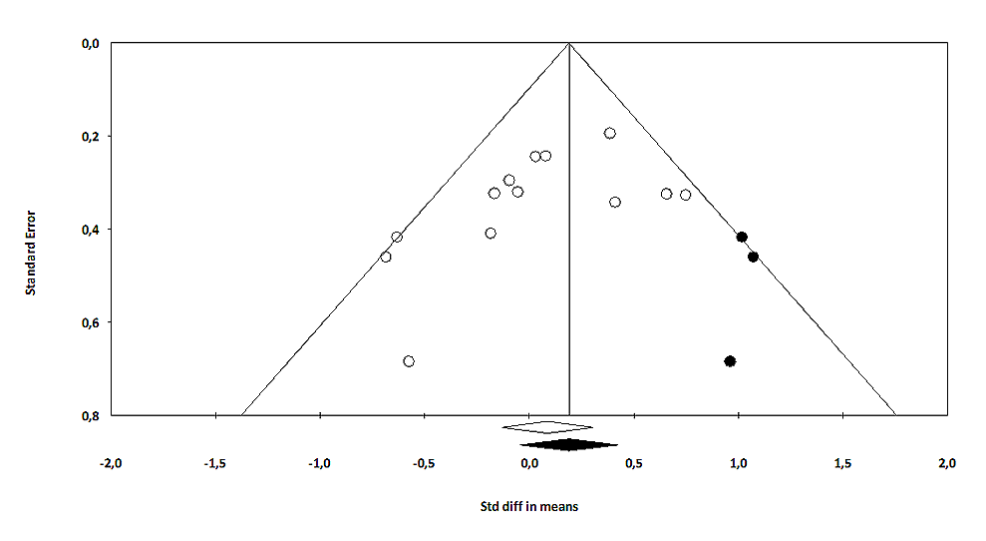
Trim and fill adjusted funnel plot of virtual reality exposure–based cognitive behavioral therapy versus cognitive behavioral therapy (the white circles represent the observed studies, and the black circles represent the imputed studies). Std diff: Standard difference.

We conducted the Egger test for asymmetry of the funnel plot for the comparison between VRE-CBT and waitlist. Visual inspection of the funnel plot seemed to show some asymmetry, which could indicate small study effects. The Egger regression intercept test was not statistically significant (intercept=−0.53, 95% CI −4.09 to 3.03; *P*=.74). The Duval and Tweedie trim and fill procedure showed that adjustment for potentially missing studies (n=1) was associated with the effect size slightly increasing from −0.51 (95% CI −0.84 to −0.17) to −0.56 (95% CI −0.88 to −0.22; Figure 5). The Egger test for the comparison between VRE with and without CBT also showed some asymmetry. The Egger regression intercept test was not statistically significant (intercept=−2.15, 95% CI −4.60 to 0.30; *P*=.08). The Duval and Tweedie trim and fill procedure showed that adjustment for potentially missing studies (n=3) was associated with the effect size increasing from 0.09 (95% CI −0.13 to 0.30) to 0.19 (95% CI −0.04 to 0.42; Figure 6), with largely overlapping CIs.

## Discussion

### Principal Findings

This is the first meta-analysis of VRE-CBT for anxiety disorders focusing solely on patients with more severe anxiety disorders with a formal diagnosis, excluding specific phobias. The meta-analysis, consisting of 16 VRE-CBT trials including 817 patients with anxiety disorders, showed a medium effect size compared with waitlist conditions. In the comparison between VRE-CBT and CBT, no significant differences were found between posttest and follow-up measurements. These findings are somewhat less positive than findings from previous meta-analyses as the effect sizes of VRE-CBT were lower in our meta-analysis. We found an effect size (Hedges *g*) of −0.49 of VRE-CBT versus waitlist compared with an estimated Hedges *g* of 0.88 and 0.79 in the meta-analyses by Carl et al [9] and Fodor et al [10], respectively. It can be hypothesized that their higher effect size is a result of the inclusion of specific phobias and subthreshold anxiety disorders. Specific phobias seemed to account for the larger effect sizes in the meta-analysis by Fodor et al [10], in which high effects of VRE-CBT for specific phobias and fight anxiety, and moderate or small effects for SAD and PTSD were found. However, in the study by Carl et al [9], this was not the case. Thus, the influence of specific phobias on the effect size is inconclusive. With regard to subthreshold anxiety disorders, neither study requested that participants be formally diagnosed with anxiety disorders. Therefore, it can be assumed that less severe cases were also included. It could be argued that people with less severe anxiety are easier to treat, and a greater effect can be reached in treatment. On the other hand, the reverse may also be true—with more symptoms at the start of treatment, greater gains can be made. Fodor et al [10] conducted sensitivity analyses that were restricted to patients with an anxiety disorder diagnosed with a clinical interview or a cutoff score on a questionnaire, which resulted in a smaller effect (Hedges *g*=0.72). This finding seems to support the hypothesis that people with less severe disorders might benefit more from treatment with VRE-CBT. With regard to the role of CBT elements or CBT sessions in 56% (9/16) of the VRE-CBT studies, sensitivity analyses with only pure VRE studies showed that pure VRE studies without additional CBT elements showed lower effect sizes in the comparison with the waitlist condition, indicating that adding CBT elements (such as psychoeducation, cognitive restructuring, interoceptive exposure, and exposure in vivo) to VRE is advised for optimizing effects.

Compared with the other studies, it is more difficult to interpret the effect size of the only study on GAD [34] in this meta-analysis as the VRE-CBT in this study mostly consisted of progressive muscle relaxation and autogenic techniques, and only 2 of 8 sessions consisted of exposure therapy. Furthermore, exposure itself differed from regular exposure-based interventions in GAD. It involved exposure to preselected words or images related to personal stressful events, although in regular CBT treatments patients with GAD are exposed to worries about hypothetical scenarios describing their worst fear [35]. It is possible that more focused exposure could lead to better results in future VRE-CBT studies for GAD.

We found no difference in attrition between VRE-CBT and CBT, which is in line with the findings of Benbow and Anderson [4], who conducted a meta-analysis on attrition between VRE-CBT and CBT across 46 studies with a combined sample size of 1057 participants. Fodor et al [10] also found that VRE-CBT yielded similar dropout rates to other interventions. These findings also suggest that VRE-CBT does not mitigate attrition.

The heterogeneity of the studies was low to moderate and did not seem to influence the results. We found no indications of small study effects or publication bias.

The risk of bias assessment in our study showed that, in many studies, it was difficult to assess risk of bias because of lacking or unclear information on random sequence generation and allocation concealment and because of lack of registration in a trial register. Keeping this in mind, it is difficult to interpret our results from the meta-regression, in which we found no significant effect of the total risk of bias score. Fodor et al [10] also used the Cochrane tool [14] for assessing the risk of bias in their meta-analysis on VRE-CBT in anxiety and depression, and they found that most studies had high or uncertain risk of bias across domains. In exploratory subgroup analyses, they also did not find differences between studies with high, uncertain, and low risk of bias, but these results must be interpreted with caution, as there were only few studies with a small risk of bias. Furthermore, previous research on adherence to study quality criteria in VRE-CBT studies on anxiety disorders by McCann et al [36] showed that the studies met an average of 2.85 (SD 1.56) of 8 quality criteria for research design, but study quality did not affect the effect size. It may be concluded that better reporting of quality in VRE-CBT RCTs is needed before conclusions can be drawn on the effect of quality on effect sizes.

### Limitations

This meta-analysis has some limitations. First, there were a limited number of trials per diagnosis (ie, none on OCD and only 1/16, 6% on GAD) and per method of recruitment (only 1/16, 6% recruited in the community) and few studies with follow-up outcome data (5/16, 31%). Subgroup analyses showed that *type of anxiety disorder* was a significant moderator in the waitlist comparison, but no conclusions can be drawn from this result, as there was high heterogeneity within the subgroups, expressing excessive clinical diversity and therefore showing no true effect. Second, we did not investigate the sense of presence or other indicators of interaction, although presence may influence the effectiveness of VRE-CBT. Ling et al [37] found a positive relationship between sense of presence and anxiety disorders, which was stronger in studies with participants with a formal anxiety disorder than in nonclinical populations. Third, 3 studies (3/45, 7%) were excluded because of missing data for analyses despite efforts to acquire these data by contacting the authors. This may have influenced the estimations.

### Clinical Implications

There are some clinical implications of the findings in this meta-analysis and for the position that VRE-CBT holds in the treatment of more severe anxiety disorders. Patients should be made aware of the state of the current evidence regarding VRE-CBT relative to alternatives to be able to make a shared decision with the therapist. The advantages of VRE-CBT over CBT may play a role in this decision. Bouchard et al [27] used a Specific Work for Exposure Applied in Therapy scale (evaluating topics such as cost, time, planning, and difficulties), which showed that VRE-CBT was more cost-efficient and practical for therapists than CBT with in vivo exposure. In addition, VRE-CBT offers a large potential for exposing patients to situations and stimuli that are too complex, costly, or challenging for in vivo exercises. It can be hypothesized that when these applications are exploited accordingly, it creates the potential to increase the effectiveness of VRE-CBT above and beyond in vivo exposure.

Furthermore, there are some specific concerns regarding VRE-CBT that clinicians should take into account: the use of the VR simulators can cause simulator sickness, appropriate training and supervision are needed, and technical support should be available in case of technical problems. In addition, although lower-cost options are becoming available, such as using smartphones as VR viewers, they still need to be evaluated [38]. Regarding improving VRE-CBT, developing VR that can be customized precisely to a patient’s needs with a better sense of presence and interaction may lead to even better outcomes.

### Conclusions

In conclusion, the results of our study show that VRE-CBT is as effective as CBT in the treatment of more severe formal anxiety disorders. However, VRE-CBT studies in this meta-analysis not only were limited in number but the quality of reporting was also poor. Therefore, awaiting further high-quality data, VRE-CBT may be considered a promising alternative to CBT, especially for patients who prefer VRE-CBT over CBT [5].

Future research should focus on conducting high-quality RCTs. Subsequently, examining for which patients or anxiety disorders VRE-CBT or CBT works best could be a next step in research.

## Appendix

Multimedia Appendix 1 Search strings for PubMed, PsycINFO, and Embase.

Multimedia Appendix 2 Study characteristics.

## Abbreviations

AGO: agoraphobia
CBT: cognitive behavioral therapy
DSM: Diagnostic and Statistical Manual of Mental Disorders
GAD: generalized anxiety disorder
ICD: International Classification of Diseases
OCD: obsessive-compulsive disorder
PD: panic disorder
PRISMA: Preferred Reporting Items for Systematic Reviews and Meta-Analyses
PTSD: posttraumatic stress disorder
SAD: social anxiety disorder
VR: virtual reality
VRE: virtual reality exposure
VRE-CBT: virtual reality exposure–based cognitive behavioral therapy

## References

[1] Olatunji BO, Cisler JM, Deacon BJ (2010). Efficacy of cognitive behavioral therapy for anxiety disorders: a review of meta-analytic findings. Psychiatr Clin North Am.

[2] Carpenter JK, Andrews LA, Witcraft SM, Powers MB, Smits JAJ, Hofmann SG (2018). Cognitive behavioral therapy for anxiety and related disorders: a meta-analysis of randomized placebo-controlled trials. Depress Anxiety.

[3] Maples-Keller JL, Bunnell BE, Kim S, Rothbaum BO (2017). The use of virtual reality technology in the treatment of anxiety and other psychiatric disorders. Harv Rev Psychiatry.

[4] Benbow AA, Anderson PL (2019). A meta-analytic examination of attrition in virtual reality exposure therapy for anxiety disorders. J Anxiety Disord.

[5] Garcia-Palacios A, Botella C, Hoffman H, Fabregat S (2007). Comparing acceptance and refusal rates of virtual reality exposure vs. in vivo exposure by patients with specific phobias. Cyberpsychol Behav.

[6] Opriş D, Pintea S, García-Palacios A, Botella C, Szamosközi Ş, David D (2012). Virtual reality exposure therapy in anxiety disorders: a quantitative meta-analysis. Depress Anxiety.

[7] Morina N, Ijntema H, Meyerbröker K, Emmelkamp PM (2015). Can virtual reality exposure therapy gains be generalized to real-life? A meta-analysis of studies applying behavioral assessments. Behav Res Ther.

[8] Powers M, Emmelkamp PM (2008). Virtual reality exposure therapy for anxiety disorders: a meta-analysis. J Anxiety Disord.

[9] Carl E, Stein A, Levihn-Coon A, Pogue JR, Rothbaum B, Emmelkamp P, Asmundson GJ, Carlbring P, Powers MB (2019). Virtual reality exposure therapy for anxiety and related disorders: a meta-analysis of randomized controlled trials. J Anxiety Disord.

[10] Fodor LA, Coteț CD, Cuijpers P, Szamoskozi Ş, David D, Cristea IA (2018). The effectiveness of virtual reality based interventions for symptoms of anxiety and depression: a meta-analysis. Sci Rep.

[11] Botella C, Fernández-Álvarez J, Guillén V, García-Palacios A, Baños R (2017). Recent progress in virtual reality exposure therapy for phobias: a systematic review. Curr Psychiatry Rep.

[12] Moher D, Liberati A, Tetzlaff J, Altman DG, PRISMA Group (2009). Preferred reporting items for systematic reviews and meta-analyses: the PRISMA statement. PLoS Med.

[13] Pitti C, Peñate W, de la Fuente J, Bethencourt J, Acosta L, Villaverde M, Gracia R (2008). [Agoraphobia: combined treatment and virtual reality. Preliminary results]. Actas Esp Psiquiatr.

[14] Higgins JP, Altman DG, Higgins JP, Green S (2008). Assessing risk of bias in included studies. Cochrane handbook for systematic reviews of interventions.

[15] Higgins JP, Thompson SG, Deeks JJ, Altman DG (2003). Measuring inconsistency in meta-analyses. BMJ.

[16] Egger M, Smith GD, Schneider M, Minder C (1997). Bias in meta-analysis detected by a simple, graphical test. BMJ.

[17] Duval S, Tweedie R (2000). Trim and fill: a simple funnel-plot-based method of testing and adjusting for publication bias in meta-analysis. Biometrics.

[18] Borenstein M, Hedges L, Higgins JP, Rothstein HR (2005). Comprehensive meta-analysis (Version 2.2.027) [Computer software]. Comprehensive meta-analysis.

[19] Liberati A, Altman DG, Tetzlaff J, Mulrow C, Gøtzsche PC, Ioannidis JP, Clarke M, Devereaux PJ, Kleijnen J, Moher D (2009). The PRISMA statement for reporting systematic reviews and meta-analyses of studies that evaluate health care interventions: explanation and elaboration. PLoS Med.

[20] Botella C, García-Palacios A, Villa H, Baños RM, Quero S, Alcañiz M, Riva G (2007). Virtual reality exposure in the treatment of panic disorder and agoraphobia: a controlled study. Clin Psychol Psychother.

[21] Choi Y, Vincelli F, Riva G, Wiederhold BK, Lee JH, Park KH (2005). Effects of group experiential cognitive therapy for the treatment of panic disorder with agoraphobia. Cyberpsychol Behav.

[22] Gonzalez LM, Peñate W, Pitti C, Bethencourt-Pére JM, de la Fuente Portero J, Marco RG (2011). Efficacy of virtual reality exposure therapy combined with two pharmacotherapies in the treatment of agoraphobia. Int J Clin Heal Psychol.

[23] Meyerbroeker K, Morina N, Kerkhof GA, Emmelkamp PM (2013). Virtual reality exposure therapy does not provide any additional value in agoraphobic patients: a randomized controlled trial. Psychother Psychosom.

[24] Peñate Castro W, Roca Sánchez MJ, Pitti González CT, Bethencourt JM, de la Fuente Portero JA, Marco RG (2014). Cognitive-behavioral treatment and antidepressants combined with virtual reality exposure for patients with chronic agoraphobia. Int J Clin Health Psychol.

[25] Pelissolo A, Zaoui M, Aguayo G, Yao SN, Roche S, Ecochard R, Gueyffier F, Pull C, Berthoz A, Jouvent R, Cottraux J (2012). Virtual reality exposure therapy versus cognitive behavior therapy for panic disorder with agoraphobia: a randomized comparison study. J Cybertherapy Rehabil.

[26] Anderson PL, Price M, Edwards SM, Obasaju MA, Schmertz SK, Zimand E, Calamaras MR (2013). Virtual reality exposure therapy for social anxiety disorder: a randomized controlled trial. J Consult Clin Psychol.

[27] Bouchard S, Dumoulin S, Robillard G, Guitard T, Klinger É, Forget H, Loranger C, Roucaut FX (2017). Virtual reality compared with in vivo exposure in the treatment of social anxiety disorder: a three-arm randomised controlled trial. Br J Psychiatry.

[28] Kampmann IL, Emmelkamp PM, Hartanto D, Brinkman WP, Zijlstra BJ, Morina N (2016). Exposure to virtual social interactions in the treatment of social anxiety disorder: a randomized controlled trial. Behav Res Ther.

[29] Moldovan R, David D (2014). One session treatment of cognitive and behavioral therapy and virtual reality for social and specific phobias. Preliminary results from a randomized clinical trial. J Evid Based Psychother.

[30] Gamito P, Oliveira J, Rosa P, Morais D, Duarte N, Oliveira S, Saraiva T (2010). PTSD elderly war veterans: a clinical controlled pilot study. Cyberpsychol Behav Soc Netw.

[31] Ready DJ, Gerardi RJ, Backscheider AG, Mascaro N, Rothbaum BO (2010). Comparing virtual reality exposure therapy to present-centered therapy with 11 U.S. Vietnam veterans with PTSD. Cyberpsychol Behav Soc Netw.

[32] Reger GM, Koenen-Woods P, Zetocha K, Smolenski DJ, Holloway KM, Rothbaum BO, Difede J, Rizzo AA, Edwards-Stewart A, Skopp NA, Mishkind M, Reger MA, Gahm GA (2016). Randomized controlled trial of prolonged exposure using imaginal exposure vs. virtual reality exposure in active duty soldiers with deployment-related posttraumatic stress disorder (PTSD). J Consult Clin Psychol.

[33] McLay RN, Wood DP, Webb-Murphy JA, Spira JL, Wiederhold MD, Pyne JM, Wiederhold BK (2011). A randomized, controlled trial of virtual reality-graded exposure therapy for post-traumatic stress disorder in active duty service members with combat-related post-traumatic stress disorder. Cyberpsychol Behav Soc Netw.

[34] Repetto C, Gaggioli A, Pallavicini F, Cipresso P, Raspelli S, Riva G (2013). Virtual reality and mobile phones in the treatment of generalized anxiety disorders: a phase-2 clinical trial. Pers Ubiquit Comput.

[35] Dugas MJ, Brillon P, Savard P, Turcotte J, Gaudet A, Ladouceur R, Leblanc R, Gervais NJ (2010). A randomized clinical trial of cognitive-behavioral therapy and applied relaxation for adults with generalized anxiety disorder. Behav Ther.

[36] McCann RA, Armstrong CM, Skopp NA, Edwards-Stewart A, Smolenski DJ, June JD, Metzger-Abamukong M, Reger GM (2014). Virtual reality exposure therapy for the treatment of anxiety disorders: an evaluation of research quality. J Anxiety Disord.

[37] Ling Y, Nefs HT, Morina N, Heynderickx I, Brinkman WP (2014). A meta-analysis on the relationship between self-reported presence and anxiety in virtual reality exposure therapy for anxiety disorders. PLoS One.

[38] Mishkind MC, Norr AM, Katz AC, Reger GM (2017). Review of virtual reality treatment in psychiatry: evidence versus current diffusion and use. Curr Psychiatry Rep.

