# Supplementary material for: The Effectiveness of Virtual Reality Exposure–Based Cognitive Behavioral Therapy for Severe Anxiety Disorders, Obsessive-Compulsive Disorder, and Posttraumatic Stress Disorder: Meta-analysis
Source: J Med Internet Res. 2022 Feb 10;24(2):e26736. doi: 10.2196/26736 (PMC8874794; doi:10.2196/26736)
Supplement: Multimedia Appendix 1 [file jmir_v24i2e26736_app1.docx]

**Appendix 1**: Search strings for Pubmed, PsycInfo, and Embase

**Pubmed**

| #22 | "Virtual Reality"[Mesh] OR "Virtual Reality Exposure Therapy"[Mesh] OR virtual realit* [tiab] OR VRET [tiab] OR VREBT [tiab] OR in virtuo exposure [tiab] OR VR treatment [tiab] OR (VR [tiab] AND exposure [tiab]) OR (VRT [tiab] AND exposure [tiab]) OR (VRE [tiab] AND exposure [tiab])) AND ("Anxiety disorders"[Mesh] OR "Stress disorders, Post-traumatic"[Mesh] OR anxiety disorder* [tiab] OR panic disorder* [tiab] OR agoraphob* [tiab] OR social phobi* [tiab] OR PTSD [tiab] OR post traumatic stress disorder* [tiab] OR posttraumatic stress disorder* OR post traumaticstress disorder* [tiab] OR obsessive compulsive disorder* [tiab] OR OCD [tiab] OR general anxiety disorder* [tiab] OR GAD [tiab] |
| --- | --- |
| #20 | "Virtual Reality"[Mesh] OR "Virtual Reality Exposure Therapy"[Mesh] OR virtual realit* [tiab] OR VRET [tiab] OR VREBT [tiab] OR in virtuo exposure [tiab] OR VR treatment [tiab] OR (VR [tiab] AND exposure [tiab]) OR (VRT [tiab] AND exposure [tiab]) OR (VRE [tiab] AND exposure [tiab]) |
| #19 | "Anxiety disorders"[Mesh] OR "Stress disorders, Post-traumatic"[Mesh] OR anxiety disorder* [tiab] OR panic disorder* [tiab] OR agoraphob* [tiab] OR social phobi* [tiab] OR PTSD [tiab] OR post traumatic stress disorder* [tiab] OR posttraumatic stress disorder* OR post traumaticstress disorder* [tiab] OR obsessive compulsive disorder* [tiab] OR OCD [tiab] OR general anxiety disorder* [tiab] OR GAD [tiab] |

**PsycInfo**

| **#** | **Query** |
| --- | --- |
| S4 | S3 |
| S3 | S1 AND S2 |
| S2 | DE "Acrophobia" OR DE "Agoraphobia" OR DE "Claustrophobia" OR DE "Ophidiophobia" OR DE "School Phobia" OR DE "Social Phobia" OR DE "Generalized Anxiety Disorder" OR DE "Obsessive Compulsive Disorder" OR DE "Panic Disorder" OR DE "Phobias" OR DE "Posttraumatic Stress Disorder" OR DE "Anxiety Disorders" OR DE "Acute Stress Disorder" OR DE "Emotional Trauma" OR DE "Post-Traumatic Stress" OR TI(“panic attack*” OR “panic disorder*” OR agoraphobi* OR acrophobi* OR claustrophobi* OR ophidiophobi* OR “obsessive compulsive disorder*" OR “social phobi*" OR PTSD OR "posttraumatic stress disorder*" OR “post-traumatic stress disorder*” OR “post-traumaticstress disorder*”OR "anxiety disorder*" OR "acute stress disorder*" OR "emotional trauma*") OR AB(“panic attack*” OR “panic disorder*” OR agoraphobi* OR acrophobi* OR claustrophobi* OR ophidiophobi* OR “obsessive compulsive disorder*" OR “social phobi*" OR PTSD OR "posttraumatic stress disorder*" OR “post-traumatic stress disorder*” OR “post-traumaticstress disorder*”OR "anxiety disorder*" OR "acute stress disorder*" OR "emotional trauma*") |
| S1 | DE "Virtual Reality" OR TI(“virtual realit*” OR VRET OR VREBT OR “in virtuo exposure” OR (VR AND exposure) OR “VR treatment”) OR AB(“virtual realit*” OR VRET OR VREBT OR “in virtuo exposure” OR (VR AND exposure) OR “VR treatment”) |

**Embase**

| **No.** | **Query** |
| --- | --- |
| #3 | #1 AND #2 |
| #2 | 'anxiety disorder'/exp OR 'panic attack*':ab,ti,kw OR 'panic disorder*':ab,ti,kw OR agoraphobi*:ab,ti,kw OR acrophobi*:ab,ti,kw OR claustrophobi*:ab,ti,kw OR ophidiophobi*:ab,ti,kw OR 'obsessive compulsive disorder*':ab,ti,kw OR 'social phobi*':ab,ti,kw OR ptsd:ab,ti,kw OR 'posttraumatic stress disorder*':ab,ti,kw OR 'post-traumatic stress disorder*':ab,ti,kw OR 'post-traumaticstress disorder*':ab,ti,kw OR 'anxiety disorder*':ab,ti,kw OR 'acute stress disorder*':ab,ti,kw OR 'emotional trauma*':ab,ti,kw |
| #1 | 'virtual reality'/exp OR 'virtual reality exposure therapy'/exp OR 'virtual realit*':ab,ti,kw OR vret:ab,ti,kw OR vrebt:ab,ti,kw OR 'in virtuo exposure':ab,ti,kw OR (vr:ab,ti,kw AND exposure:ab,ti,kw) OR 'vr treatment':ab,ti,kw |
