# Supplementary material for: The Effectiveness of Virtual Reality Exposure–Based Cognitive Behavioral Therapy for Severe Anxiety Disorders, Obsessive-Compulsive Disorder, and Posttraumatic Stress Disorder: Meta-analysis
Source: J Med Internet Res. 2022 Feb 10;24(2):e26736. doi: 10.2196/26736 (PMC8874794; doi:10.2196/26736)
Supplement: Multimedia Appendix 2 [file jmir_v24i2e26736_app2.docx]

**Appendix 2. Study characteristics.**

| Study | | Total randomized participants, n | Age (years), mean (standard deviation, range)^a^ | Women, n (%) | Diagnosis method (instrument) | Recruitment | Primary outcome | ITT^b^ | Comparisons (treatment components: VRE^c^, CBT^d^, and WL^e^) | Participants, n | Dropouts, n | Sessions, n | Follow-up |
| --- | --- | --- | --- | --- | --- | --- | --- | --- | --- | --- | --- | --- | --- |
| **Panic disorder with or without agoraphobia** | | | | | | | | | | | | | |
|  | Botella et al [20] | 37 | 34.7 (12.3, 18-72) | 26 (70) | DSM-IV^f^ (ADIS-IV^g^) | Community + clinical | PDSS^h^ | No | VRE + CBT, CBT, and WL | VRE + CBT and CBT: 12; WL: 13 | VRE + CBT, CBT, and WL: NR^i^ | VRE + CBT and CBT: 9; WL: —^j^ | VRE + CBT and CBT: 12 months; WL: — |
|  | Choi et al [21] | 40 | NA | 20 (50) | DSM-IV (as diagnosed by 2 psychiatrists) | Clinical | ACQ^k^ | No | VRE + CBT and CBT | VRE + CBT and CBT: 20 | VRE + CBT and CBT: NR | VRE + CBT: 4; CBT: 12 | VRE + CBT and CBT: No |
|  | González Lorenzo et al [22] | 64 | NA | NA | DSM-IV and ICD-10^l^ (CIDI^m^) | Clinical | ACQ | No | VRE + CBT, CBT, and WL^n^ | VRE + CBT and CBT: 22; WL: 20 | VRE + CBT: 3; CBT: 2; WL: 0 | VRE + CBT and CBT: 11; WL: — | VRE + CBT and CBT: 6 months; WL: — |
|  | Meyerbroeker et al [23] | 55 | NR (NR, 18-65) | NR | DSM-IV (SCID^o^) | NR | ACQ | Yes | VRE + CBT and CBT | VRE + CBT: 24; CBT: 22 | VRE + CBT: 9; CBT: 8 | VRE + CBT and CBT: 10 | VRE + CBT and CBT: No |
|  | Penate-Castro et al [24] | 80 | NA | NA | DSM-IV-TR^p^ and ICD-10 (CIDI) | Clinical | ACQ | Yes | VRE, CBT, and WL | VRE and CBT: 30; WL: 20 | VRE: 7; CBT: 16; WL: 7 | VRE and CBT: 11; WL: — | VRE and CBT: 6 months; WL: — |
|  | Pelissolo et al [25] | 92 | NA) | 62 (67) | DSM-IV (MINI^q^) | Clinical | FQ^r^ (ss AF^s^) | No | VRE + CBT and CBT | VRE + CBT: 33; CBT: 34 | VRE + CBT: 10; CBT: 7 | VRE + CBT and CBT: 12 | VRE + CBT and CBT: 9 months |
|  | Pitti et al [13] | 27 | 38.5 (9.3, NR) | 22 (81) | ICD-10 (CIDI) | Clinical | ACQ | No | VRE + CBT and CBT | VRE + CBT: 18; CBT: 9 | VRE + CBT and CBT: NR | VRE + CBT and CBT: 11 | VRE + CBT and CBT: No |
| **Social anxiety disorder** | | | | | | | | | | | | | |
|  | Anderson et al [26] | 97 | 39.0 (11.3, 19-69) | 60 (62) | DSM-IV-TR (SCID) | Community + clinical | FNE-B^t^ | Yes | VRE, CBT, and WL | VRE: 30; CBT: 39; WL: 28 | VRE: 5; CBT: 14; WL: 3 | VRE and CBT: 8; WL: — | VRE and CBT: 12 months; WL: — |
|  | Bouchard et al [27] | 59 | 34.5 (NR, NR) | 43 (73) | DSM-IV (SCID) | Community + clinical | LSAS-SR^u^ | Yes | VRE + CBT, CBT, and WL | VRE + CBT: 17; CBT: 22; WL: 20 | VRE + CBT, CBT, and WL: 4 | VRE + CBT and CBT: 14; WL: — | VRE + CBT and CBT: 6 months; WL: — |
|  | Kampmann et al^v^[28] | 60 | 36.9 (NR, 18-65) | 38 (63) | DSM-IV (SCID-I) | Community | LSAS-SR | Yes | VRE, CBT, and WL | VRE, CBT, and WL: 20 | VRE: 5; CBT: 3; WL: 2 | VRE and CBT: 10; WL: — | VRE and CBT: 3 months; WL: — |
|  | Moldovan and David^w^[29] | 15 | NR (NR) | 9 (60) | DSM-IV (SCID) | NR | LSAS | No | VRE + CBT and WL | VRE + CBT: 7; WL: 5 | VRE + CBT and WL: 0 | VRE + CBT: 1; WL: — | VRE + CBT: No; WL: — |
| **Posttraumatic stress disorder** | | | | | | | | | | | | | |
|  | Gamito et al [30] | 10 | 63.5 (4.4, NR) | 0 (0) | DSM-IV (CAPS^x^) | NR | CAPS | No | VRE and WL | VRE: 5; WL: 3 | VRE: 1; WL: 0 | VRE: 12; WL: — | VRE: No; WL: — |
|  | Ready et al [31] | 11 | 57.5 (NR, 53-62) | 0 (0) | DSM-IV (CAPS) | Community | CAPS | No | VRE and PCT^y^ | VRE: 6; PCT: 5 | VRE and PCT: 1 | VRE and PCT: 10 | VRE: 6 months; PCT: — |
|  | Reger et al [32] | 162 | 30.3 (NR, NR) | 6 (4) | DSM-IV-TR (CAPS) | Community + clinical | CAPS | Yes | VRE, CBT, and WL | VRE, CBT, and WL: 54 | VRE: 5; CBT and WL: 7 | VRE and CBT: 10; WL: — | VRE and CBT: 6 months; WL: — |
|  | McLay et al [33] | 20 | 28.4 (NR, 21-45) | 1 (5) | DSM-IV (MINI, CAPS) | Community + clinical | CAPS | No | VRE and TAU/CBT^z^ | VRE and TAU/CBT: 10 | VRE: 0; TAU/CBT: 1 | VRE: 12.3 (mean); TAU/CBT: 13.8 (mean) | VRE and TAU/CBT: No |
| **Generalized anxiety disorder** | | | | | | | | | | | | | |
|  | Repetto et al [34] | 25 | 47.6 (NR) | 16 (64) | DSM-IV-TR, interview (NR) | Clinical | HAM-A^aa^ | No | VRE^bb^ and WL | VRE: 17; WL: 8 | VRE: 1; WL: 0 | VRE: 8; WL: — | VRE: No; WL: — |

^a^If reported for the sample of total randomized participants in the original article.

^b^ITT: intention-to-treat.

^c^VRE: virtual reality exposure.

^d^CBT: cognitive behavioral therapy. CBT entailed exposure in vivo, prolonged exposure, exposure in imagination, and/or cognitive restructuring.

^e^WL: waitlist.

^f^DSM-IV: Diagnostic and Statistical Manual of Mental Disorders, Fourth Edition.

^g^ADIS-IV: Anxiety Disorders Interview Schedule for the DSM-IV.

^h^PDSS: Panic Disorder Severity Scale.

^i^NR: not reported.

^j^NA: not applicable.

^k^ACQ: Agoraphobic Cognitions Questionnaire.

^l^ICD-10: International Classification of Diseases, 10th revision.

^m^CIDI: Composite International Diagnostic Interview.

^n^For each condition (VRE, CBT, and WL), 2 medication groups were pooled (paroxetine or venlafaxine).
^o^SCID: Structured Clinical Interview for the DSM-IV.
^p^DSM-IV-TR: Diagnostic and Statistical Manual of Mental Disorders, 4th Edition, Text Revision.

^q^Mini-International Neuropsychiatric Interview.

^r^FQ: Fear Questionnaire.

^s^ss AF: Subscale Agoraphobia.

^t^FNE-B: The Fear of Negative Evaluation-Brief Form.

^u^LSAS-SR: Liebowitz Social Anxiety Scale-Self Report.

^v^Unpooled data were used.

^w^Crossover patients were excluded from the postanalysis data to maintain the original randomization.

^x^CAPS: Clinician-Administered Posttraumatic Stress Disorder (PTSD) Scale.

^y^PCT: Present-Centered Therapy. PCT is a supportive therapy that includes psychoeducation about PTSD and teaching problem-solving techniques (active control).

^z^TAU/CBT: treatment as usual, including a full spectrum of PTSD treatment (active control).

^aa^HAM-A: Hamilton Anxiety Rating Scale.

^bb^For the virtual reality condition, 2 virtual reality groups (with and without biofeedback) were pooled.

20. Botella C, Gracía-Palacios A, Villa H, Baños RM, Quero S, Alcañiz M, et al. Virtual reality exposure in the treatment of panic disorder and agoraphobia: A controlled study. Clin Psychol Psychother.; 2007;14(3):164–175.

21. Choi YH, Vincelli F, Riva G, Wiederhold BK, Lee JH, Park KH. Effects of group experiential cognitive therapy for the treatment of panic disorder with agoraphobia. Cyberpsychology Behav. 2005;8(4):387–393. PMID: 16092896

22. Gonzalez Lorenzo M, Peñate W, Pitti C, Bethencourt-Pére J, Portero J, Marco R. Efficacy of virtual reality exposure therapy combined with two pharmacotherapies in the treatment of agoraphobia. Int J Clin Heal Psychol. 2011;11:189–203.

23. Meyerbroeker K, Morina N, Kerkhof GA, Emmelkamp PMG. Virtual reality exposure therapy does not provide any additional value in agoraphobic patients: A randomized controlled trial. Psychother Psychosom.; 2013;82(3):170–176. PMID: 23548832

24. Penate-Castro WP, Sánchez MJR, González CTP, Bethencourt JM, de la Fuente Portero JA, Marco RG. Cognitive-behavioral treatment and antidepressants combined with virtual reality exposure for patients with chronic agoraphobia. Int J Clin Heal Psychol. Elsevier; 2014;14(1):9–17.

25. Pelissolo A, Zaoui M, Aguayo G, Yao SN, Roche S, Ecochard R, et al. Virtual reality exposure therapy versus cognitive behavior therapy for panic disorder with agoraphobia: a randomized comparison study. J Cybertherapy Rehabil. 2012;5(1):35–43.

13. Pitti C, Peñate W, de la Fuente J, Bethencourt J, Acosta L, Villaverde M, et al. [Agoraphobia: combined treatment and virtual reality. Preliminary results]. Actas Esp Psiquiatr.; 2008;36(2):94–101. PMID: 18365789

26. Anderson PL, Price M, Edwards SM, Obasaju MA, Schmertz SK, Zimand E, et al. Virtual reality exposure therapy for social anxiety disorder: A randomized controlled trial. J Consult Clin Psychol.; 2013;81(5):751–760. PMID: 23796315

27. Bouchard S, Dumoulin S, Robillard G, Guitard T, Klinger E, Forget H, et al. Virtual reality compared with in vivo exposure in the treatment of social anxiety disorder: A three-arm randomised controlled trial. Br J Psychiatry; 2017;210(4):276–283. PMID: 27979818

28. Kampmann IL, Emmelkamp PMG, Hartanto D, Brinkman WP, Zijlstra BJH, Morina N. Exposure to virtual social interactions in the treatment of social anxiety disorder: A randomized controlled trial. Behav Res Ther.; 2016;77:147–156. PMID: 26752328

29. Moldovan R, David D. One session treatment of cognitive and behavioral therapy and virtual reality for social and specific phobias. Preliminary results from a randomized clinical trial. J Evid Based Psychother. 2014;XIV:67–83.

30. Gamito P, Oliveira J, Rosa P, Morais D, Duarte N, Oliveira S, et al. PTSD elderly war veterans: A clinical controlled pilot study. Cyberpsychology, Behav Soc Netw.; 2010;13(1):43–48. PMID: 20528292

31. Ready DJ, Gerardi RJ, Backscheider AG, Mascaro N, Rothbaum BO. Comparing virtual reality exposure therapy to present-centered therapy with 11 U.S. Vietnam veterans with PTSD. Cyberpsychology, Behav Soc Netw. D.J.; 2010;13(1):49–54. PMID: 20528293

32. Reger GM, Koenen-Woods P, Zetocha K, Smolenski DJ, Holloway KM, Rothbaum BO, et al. Randomized controlled trial of prolonged exposure using imaginal exposure vs. virtual reality exposure in active duty soldiers with deployment-related posttraumatic stress disorder (PTSD). J Consult Clin Psychol.; 2016;84(11):946–959. PMID: 27606699

33. McLay RN, Wood DP, Webb-Murphy JA, Spira JL, Wiederhold MD, Pyne JM, et al. A randomized, controlled trial of virtual reality-graded exposure therapy for post-traumatic stress disorder in active duty service members with combat-related post-traumatic stress disorder. Cyberpsychology, Behav Soc Netw. R.N.; 2011;14(4):223–229. PMID: 21332375

34. Repetto C, Gaggioli A, Pallavicini F, Cipresso P, Raspelli S, Riva G. Virtual reality and mobile phones in the treatment of generalized anxiety disorders: a phase-2 clinical trial. Pers Ubiquit Comput. 2013;17:253–260.
